# Lymphovascular invasion and p16 expression are independent prognostic factors in stage I vulvar squamous cell carcinoma

**DOI:** 10.1007/s00428-023-03670-y

**Published:** 2023-10-16

**Authors:** Ben Davidson, Tone Skeie-Jensen, Arild Holth, Kristina Lindemann, Anne Marie Toralba Barrameda, Agnes Kathrine Lie, Yun Wang

**Affiliations:** 1 Department of Pathology, Norwegian Radium Hospital, Oslo University Hospital, Montebello, N-0310 Oslo, Norway; 2 Faculty of Medicine, Institute of Clinical Medicine, University of Oslo, N-0316 Oslo, Norway; 3 Department of Gynecologic Oncology, Norwegian Radium Hospital, Oslo University Hospital, Montebello, N-0310 Oslo, Norway

**Keywords:** Vulva, Squamous cell carcinoma, p16, p53, HPV, Prognostic

## Abstract

The objective of this study was to identify clinicopathologic parameters associated with disease outcome in FIGO stage I vulvar squamous cell carcinoma (vSqCC). The cohort consisted of 126 patients diagnosed with vSqCC in the period 2006–2016 who underwent primary vulvar surgery and evaluation of groin lymph node status. Tumors were reviewed by an experienced gynecologic pathologist. p16 and p53 protein expression by immunohistochemistry and HPV status were analyzed in 116 tumors. Clinicopathologic parameters, protein expression and HPV status were analyzed for association with progression-free and overall survival (PFS, OS). p16 expression and aberrant p53 were found in 49 (42%) and 61 (53%) tumors, respectively. Sixty-six tumors were HPV-associated (57%). Relapse was diagnosed in 35/126 (28%) of patients, and 23 (18%) died of disease. Tumor diameter > 4 cm (*p* = 0.013), lymphovascular space invasion (LVSI; *p* < 0.001), the presence of lichen sclerosus (*p* = 0.019), p16 expression (*p* = 0.007), p53 expression (*p* = 0.012), HPV status (*p* = 0.021), lymph node metastasis (*p* < 0.001) and post-operative radiotherapy (*p* < 0.001) were significantly related to OS in univariate analysis. Tumor diameter > 4 cm (*p* = 0.038), LVSI (*p* = 0.003), the presence of lichen sclerosus (*p* = 0.004), p16 expression (*p* = 0.004), HPV status (*p* = 0.039), lymph node metastasis (*p* < 0.001) and post-operative treatment (*p* < 0.001), were significantly related to PFS in univariate analysis. Age, BMI and surgical resection involvement were not significantly associated with OS or PFS. In multivariate Cox analysis, LVSI and p16 expression were independent prognosticators of OS (*p* < 0.001 and *p* = 0.02, respectively) and PFS (*p* = 0.018, *p* = 0.037). In conclusion, LVSI and p16 expression are independent prognostic factors in stage I vSqCC.

**Supplementary Information:**

The online version contains supplementary material available at 10.1007/s00428-023-03670-y.

## Introduction

Vulvar cancer is a rare malignancy, with 45,240 cases diagnosed globally in 2020, and 17,427 cancer-related death during this year, both consisting 0.2% of the entire cancer burden [1]. The majority (> 90%) of vulvar cancers are squamous cell carcinomas (vSqCC), consisting of HPV-associated and HPV-independent tumors. The former have as precursor high-grade vulvar intraepithelial neoplasia (HG-VIN), while in the latter are associated with differentiated VIN (dVIN) and lichen sclerosus (LS). HPV16 is the most common type found in HPV-associated carcinomas, whereas *TP53* mutations are common in HPV-independent carcinomas. Immunostaining for p16 and p53 has been applied as surrogate markers in HPV-associated and HPV-independent carcinomas, respectively. Surgery is the mainstay of treatment for vulvar cancer, with adjuvant radiotherapy applied in some cases. Five-year survival is estimated at 50–70%, and is worse for patients with HPV-independent tumors [2].

Clinicopathologic parameters that have been assessed for potential association with survival include the type of precursor lesion present (HG-VIN vs. dVIN), presence of LS, histological grade, tumor size, depth of invasion, stromal changes, pattern of invasion, lymphovascular space invasion (LVSI), perineural invasion, tumor focality, resection margin status, lymph node metastasis, HPV status/p16 expression, patient age and tumor stage. Results have been variable, with tumor stage and lymph node status shown most consistently to be prognosticators in this disease [reviewed in 3].

The objective of the present study was to analyze the role of clinicopathologic parameters in a Norwegian cohort of patients diagnosed with stage I vSqCC.

## Materials and methods

### Study population

The study cohort consisted of 126 patients diagnosed with stage I vSqCC without clinical, radiological, or cytological evidence of groin lymph node metastasis at the time of primary diagnosis, who were treated at the Department of Gynecological Oncology, Oslo University Hospital – Norwegian Radium Hospital between 01.01.2006 and 31.12.2016. All patients underwent radical wide local excision of the primary tumor, in combination with unilateral or bilateral sentinel lymph node dissection (SLND) or inguinofemoral lymphadenectomy. In unilateral tumors, only ipsilateral lymphadenectomy was performed. Since 2009, SLND has been the standard of care for unifocal tumors smaller than 4 cm at our institution.

All specimens underwent histopathological review by a surgical pathologist specialized in gynecologic pathology (BD), who confirmed the diagnosis and assessed resection margin status, and the presence of lichen sclerosus (LS) and lymphovascular space invasion (LVSI). In agreement with the recommendations from the International Collaboration on Cancer Reporting (ICCR) [4], tumors were not graded.

Patients with positive sentinel node or positive surgical margin/close distance to tumor-free margin underwent either re-operation or received adjuvant radiotherapy if re-operation is not possible, with or without concomitant weekly cisplatin, with a dose up to 70 Gy. Insufficient pathological tumor-free margin distance was defined as < 8 mm [5]. When re-excision was performed, the closest margin after re-excision was assessed. After primary treatment, patients were examined routinely every three months during the first two years, every six months during the third to fifth year, and annually thereafter.

Information on baseline clinicopathologic characteristics, treatment, recurrence and follow-up was obtained from the patients’ electronic records. Individual survival data were available through linkage to Statistics Norway. In the present study, the following 10 clinicopathologic factors were analyzed as possible risk factors for tumor recurrence: Age at diagnosis, tumor size, resection margin status, the presence of LS or LVSI, p53 status, p16 expression, the presence of HPV, groin lymph node metastasis and administration of adjuvant radiotherapy.

### Immunohistochemistry (IHC)

Formalin-fixed, paraffin-embedded sections from 116 tumors with available block were analyzed for p16 and p53 protein expression using the Dako EnVision Flex + System (K8012; Dako, Glostrup, Denmark). The p16 antibody was a mouse monoclonal antibody purchased from NeoMarkers/Thermo Fisher Scientific Inc. (cat # MS-887-P; clone 16P04; Fremont, CA), applied at a 1:500 dilution following antigen retrieval in LpH buffer (pH 6.0). The p53 antibody was a mouse monoclonal antibody purchased from Santa Cruz Biotechnology (cat # sc-126; clone DO-1; Santa Cruz, CA), applied at a 1:500 dilution following antigen retrieval in HpH buffer (pH 9.0).

Following deparaffinization, sections were treated with EnVision™ Flex + mouse linker (15 min) and EnVision™ Flex/HRP enzyme (30 min) and stained for 10 min with 3′3-diaminobenzidine tetrahydrochloride (DAB), counterstained with hematoxylin, dehydrated and mounted in Toluene-Free Mounting Medium (Dako). Positive control for p16 and p53 consisted of high-grade serous carcinoma and colon carcinoma, respectively. In negative controls, the primary antibody was replaced with isotype-specific mouse myeloma protein diluted to the same concentration as the primary antibody.

#### IHC scoring

Staining was scored by a gyn-pathologist (BD). p16 expression was scored as diffuse, patchy or absent, the latter two grouped as negative. p53 expression was scored as wild-type vs. aberrant (mutation-type) based on recent recommendations [6].

### HPV typing

HPV status was analyzed in 116 tumors with available block. Tumor tissue was highlighted on the H&E slide by the study pathologist prior to DNA extraction. Four 10 µm-thick sections were cut for DNA extraction. Between each patient block, the microtome was cleaned with RNase Zap (decontamination solution), distilled water, and 70% ethanol; gloves and the microtome blade were changed, and a control block, containing only paraffin, was sectioned.

Total DNA was extracted using the QIAamp DNA FFPE Tissue Kit (Qiagen GmbH, Hilden, Germany). Tumor tissue corresponding to the marked H&E sections was scraped and paraffin was removed in 160µL deparaffinization solution (Qiagen). Tissue was then lysed at 56 °C overnight with 20µL proteinase K. DNA was subsequently purified in several wash steps according to the manufacturer guidelines, eluted in 90µL ATE buffer, and stored at 4 °C until further use.

HPV DNA detection was performed with the AnyplexTMII HPV28 Detection Kit (Seegene, Seoul, South Korea) as described by the manufacturer. The assay detects 28 different HPV genotypes, including high-risk (16, 18, 31, 33, 35, 39, 45, 51, 52, 56, 58 and 59), probably or possibly high-risk (68, 26, 53, 66, 67, 70, 73 and 82) and low-risk genotypes (6, 11, 40, 42, 43, 44, 54 and 61), based on current guidelines [7, 8].

### Statistical analysis

Statistical analysis was performed applying the SPSS-PC package (Version 28). Probability of < 0.05 was considered statistically significant.

For progression free survival (PFS), follow-up time was calculated from the date of diagnosis until the date of relapse, date of death from any cause or end of follow-up (15.02.20). For overall survival (OS), follow-up time was calculated from the date of diagnosis until date of death from any cause or end of follow-up, whichever occurred first. Survival curves were plotted with the Kaplan–Meier method. The log rank test was used to compare survival between the groups. Multivariate survival analysis was performed using Cox proportional hazard models.

## Results

**Fig. 1:**
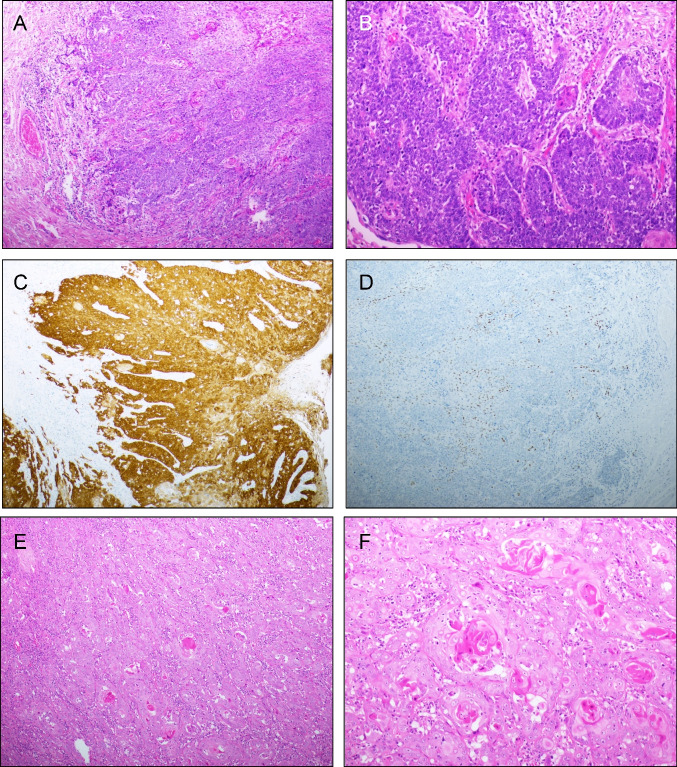

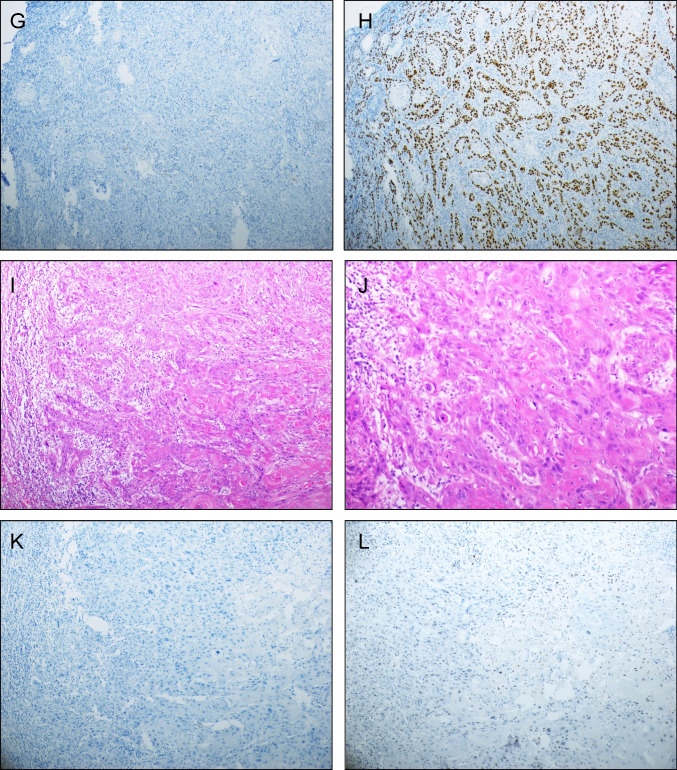
IHC. (**A-D**) HPV-associated SqCC. The tumor is diffusely positive for p16 (**C**) and has wild-type p53 staining pattern (**D**). HPV analysis was positive for HPV16 DNA. (**E–H**) HPV-independent SqCC. The tumor is negative for p16 (**G**) and has aberrant (mutation-type) p53 staining pattern (**H**). HPV analysis was negative. (**I-L**) HPV-independent SqCC. This tumor is negative for p16 (K) and has wild-type p53 staining pattern (**L**). HPV analysis was negative

Clinicopathologic data, as well as IHC and HPV typing results are summarized in Table 1. p16 and p53 immunostaining is illustrated in Fig. 1.

**Table 1:** Clinicopathologic parameters of the cohort (126 patients)

Parameter	Distribution
Age (mean)	29–93 years (63)
Tumor diameter	
≤ 4 cm	102 (81%)
> 4 cm	24 (19%)
Lymphovascular invasion	
Yes	17 (13%)
No	108 (86%)
NA	1 (1%)
Lichen sclerosus	
Yes	37 (29%)
No	87 (69%)
NA	2 (2%)
Pathological tumor-free margin	
Yes	108 (86%)
No	9 (7%)
NA	9 (7%)
Lymph node metastasis	
Yes	30 (24%)
No	84 (67%)
NA	12 (9%)
p16	
Positive	49 (39%)
Patchy/negative	63 (50%)
Inconclusive	4 (3%)
NA	10 (8%)
p53	
Aberrant	61 (48%)
Wild-type	55 (44%)
NA	10 (8%)
HPV status	
Positive	66 (52%)
Negative	49 (39%)
NA	11 (9%)
Post-operative radiation	
Yes	29 (23%)
No	97 (77%)
Relapse	
Yes	35 (28%)
No	91 (72%)
Status	
NED	78 (62%)
DOD	23 (18%)
DOOC	25 (20%)

*Abbreviations*: *NA* not available, *NED* no evidence of disease, *DOD* dead of disease, *DOOC* dead of other cause

HPV16 was found in 54 of the 66 (82%) HPV-positive tumors, including 48 tumors in which it was the single virus type and 6 in which mixed infection was found. The remaining cases harbored HPV33 (7 tumors), HPV18 (2 tumors), HPV51 (1 tumor), HPV73 (1 tumor) and mixed infection with HPV types 42, 61 and 6 (1 case).

Positive p16 expression and aberrant p53 were mutually exclusive in the majority (93/116; 80%) of cases (42 p16-positive tumors and 51 p53-aberrant tumors), whereas in the remaining tumors both analyses were positive (7 cases) or negative (12 cases). In 4 tumors assessment was impossible due to equivocal p16 expression. HPV status and p16 expression correlated in 91/115 (79%) tumors. The remaining tumors consisted of 18 p16-negative, HPV-positive and 2 p16-positive, HPV-negative cases, as well as the 4 above-mentioned cases in which p16 staining could not be classified with certainty.

The follow-up period ranged from 5 to 165 months (mean = 71 months, median = 67 months). Relapse was diagnosed in 35/126 (28%) of patients, and 23 (18%) died of disease.

**Fig. 2:**
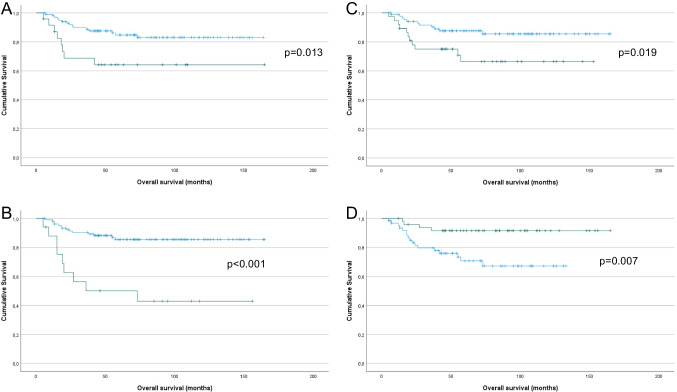

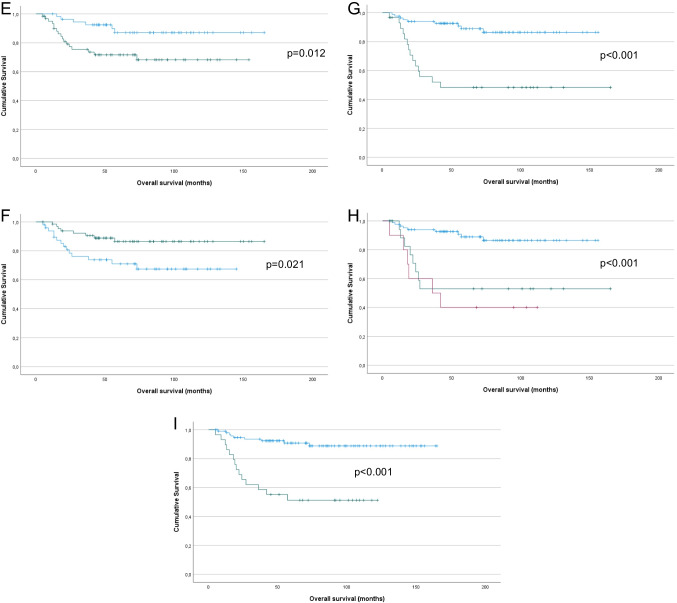
Survival analysis – OS. **A:** Kaplan–Meier survival curve showing the association between tumor diameter and OS for 126 vulvar SqCC patients. Patients with tumor diameter > 4 cm (*n* = 24; green line) had mean OS of 112 months compared to 142 months for patients with tumor diameter > 4 cm (*n* = 102, blue line; *p* = 0.013). **B:** Kaplan–Meier survival curve showing the association between the presence of LVSI and OS for 125 vulvar SqCC patients (1 patient with missing data). Patients with LVSI (*n* = 17; green line) had mean OS of 81 months compared to 145 months for patients whose tumors did not have LVSI (*n* = 108, blue line; *p* < 0.001). **C:** Kaplan–Meier survival curve showing the association between the presence of LS and OS for 124 vulvar SqCC patients (2 patients with missing data). Patients with LS (*n* = 37; green line) had mean OS of 110 months compared to 146 months for patients whose tumors did not have LS (*n* = 87, blue line; *p* = 0.019). **D:** Kaplan–Meier survival curve showing the association between p16 expression and OS for 112 vulvar SqCC patients with conclusive data (10 tumors not analyzed, 4 tumors with equivocal staining). Patients with p16-positive tumors (*n* = 49; green line) had mean OS of 153 months compared to 100 months for patients with p16-negative tumors (*n* = 63, blue line; *p* = 0.007). **E:** Kaplan–Meier survival curve showing the association between p53 expression and OS for 116 vulvar SqCC patients (10 tumors not analyzed). Patients with tumors that had aberrant p53 staining pattern (*n* = 61; green line) had mean OS of 113 months compared to 148 months for patients with wild-type staining pattern (*n* = 55, blue line; *p* = 0.012). **F:** Kaplan–Meier survival curve showing the association between HPV status and OS for 115 vulvar SqCC patients (10 tumors not analyzed, 1 failed test). Patients with HPV-positive tumors (*n* = 66; green line) had mean OS of 147 months compared to 107 months for patients with HPV-negative tumors (*n* = 49, blue line; *p* = 0.021). **G:** Kaplan–Meier survival curve showing the association between lymph node metastasis (yes vs. no) and OS for 114 vulvar SqCC patients (12 patients with no data). Patients with lymph node metastasis (*n* = 30; green line) had mean OS of 91 months compared to 140 months for patients with no metastasis (*n* = 84, blue line; *p* < 0.001). **H:** Kaplan–Meier survival curve showing the association between lymph node metastasis (0 vs. 1 vs. > 1) and OS for 114 vulvar SqCC patients (12 patients with no data). Patients with > 1 (*n* = 10; red line) and 1 lymph node metastasis (*n* = 20; green line) had mean OS of 58 and 97 months, respectively, compared to 140 months for patients with no metastasis (*n* = 84, blue line; *p* < 0.001). **I:** Kaplan–Meier survival curve showing the association between post-operative radiotherapy and OS for 126 vulvar SqCC patients. Patients who received post-operative radiotherapy (*n* = 29; green line) had mean OS of 74 months compared to 150 months for patients who did not require post-operative radiotherapy (*n* = 97, blue line; *p* < 0.001)

In univariate analysis of OS, tumor diameter > 4 cm (Fig. 2A; *p* = 0.013), LVSI (Fig. 2B; *p* < 0.001), the presence of LS (Fig. 2C; *p* = 0.019), negative p16 expression (Fig. 2D; *p* = 0.007), aberrant p53 expression (Fig. 2E; *p* = 0.012), negative HPV status (Fig. 2F; *p* = 0.021), lymph node metastasis (yes vs. no and 0 vs. 1 vs. > 1, Fig. 2G and H, respectively; both *p* < 0.001) and post-operative radiotherapy (Fig. 2I; *p* < 0.001) were significantly related to shorter OS. Perinodal involvement was additionally significantly associated with shorter OS (*p* = 0.029), though the number of events (= 5) was deemed too small for meaningful analysis (data not shown).

**Fig. 3:**
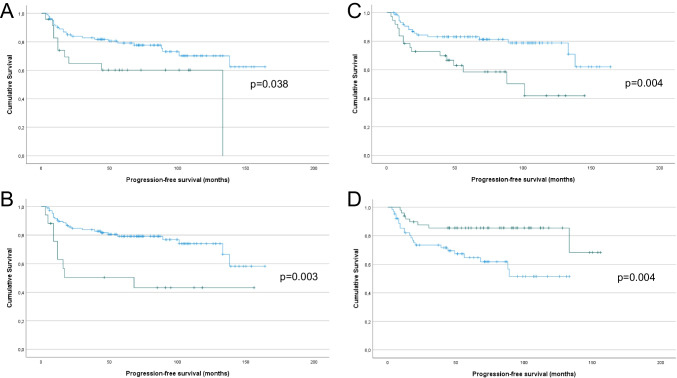

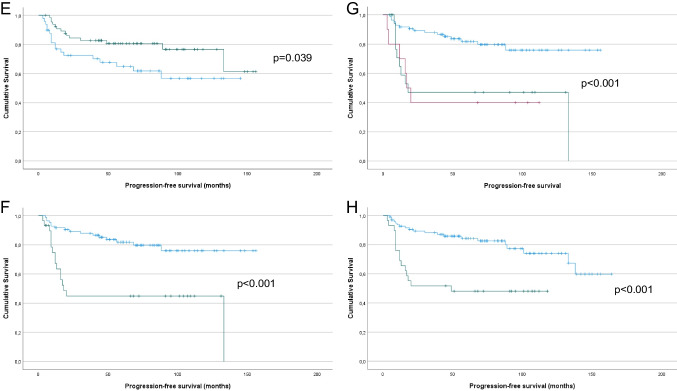
Survival analysis – PFS. **A:** Kaplan–Meier survival curve showing the association between tumor diameter and PFS for 126 vulvar SqCC patients. Patients with tumor diameter > 4 cm (*n* = 24; green line) had mean PFS of 86 months compared to 125 months for patients with tumor diameter > 4 cm (*n* = 102, blue line; *p* = 0.038). **B:** Kaplan–Meier survival curve showing the association between the presence of LVSI and PFS for 125 vulvar SqCC patients (1 patient with missing data). Patients with LVSI (*n* = 17; green line) had mean PFS of 76 months compared to 126 months for patients whose tumors did not have LVSI (*n* = 108, blue line; *p* = 0.003). **C:** Kaplan–Meier survival curve showing the association between the presence of LS and PFS for 124 vulvar SqCC patients (2 patients with missing data). Patients with LS (*n* = 37; green line) had mean PFS of 86 months compared to 130 months for patients whose tumors did not have LS (*n* = 87, blue line; *p* = 0.004). **D:** Kaplan–Meier survival curve showing the association between p16 expression and PFS for 112 vulvar SqCC patients with conclusive data (10 tumors not analyzed, 4 tumors with equivocal staining). Patients with p16-positive tumors (*n* = 49; green line) had mean PFS of 132 months compared to 87 months for patients with p16-negative tumors (*n* = 63, blue line; *p* = 0.004). **E:** Kaplan–Meier survival curve showing the association between HPV status and PFS for 115 vulvar SqCC patients (10 tumors not analyzed, 1 failed test). Patients with HPV-positive tumors (*n* = 66; green line) had mean PFS of 123 months compared to 95 months for patients with HPV-negative tumors (*n* = 49, blue line; *p* = 0.039). **F:** Kaplan–Meier survival curve showing the association between lymph node metastasis (yes vs. no) and PFS for 114 vulvar SqCC patients (12 patients with no data). Patients with lymph node metastasis (*n* = 30; green line) had mean PFS of 66 months compared to 128 months for patients with no metastasis (*n* = 84, blue line; *p* < 0.001). **G:** Kaplan–Meier survival curve showing the association between lymph node metastasis (0 vs. 1 vs. > 1) and PFS for 114 vulvar SqCC patients (12 patients with no data). Patients with > 1 (*n* = 10; red line) and 1 lymph node metastasis (*n* = 20; green line) had mean OS of 52 and 69 months, respectively, compared to 128 months for patients with no metastasis (*n* = 84, blue line; *p* < 0.001). **H:** Kaplan–Meier survival curve showing the association between post-operative radiotherapy and PFS for 126 vulvar SqCC patients. Patients who received post-operative radiotherapy (*n* = 29; green line) had mean PFS of 64 months compared to 130 months for patients who did not require post-operative radiotherapy (*n* = 97, blue line; *p* < 0.001)

In univariate analysis of PFS, tumor diameter > 4 cm (Fig. 3A; *p* = 0.038), LVSI (Fig. 3B; *p* = 0.003), the presence of LS (Fig. 3C; *p* = 0.004), negative p16 expression (Fig. 3D; *p* = 0.004), negative HPV status (Fig. 3E; *p* = 0.039), lymph node metastasis (yes vs. no and 0 vs. 1 vs. > 1, Fig. 3F and G, respectively; both *p* < 0.001) and post-operative radiotherapy (Fig. 3H; *p* < 0.001) were significantly related to shorter PFS. p53 expression was associated with only a trend for shorter PFS (*p* = 0.058).

Age, BMI and surgical resection involvement by VIN or SqCC were not significantly associated with OS or PFS (*p* > 0.05).

In Cox multivariate analysis, LVSI and p16 expression were independent prognosticators of OS (*p* < 0.001 and *p* = 0.02, respectively) and PFS (*p* = 0.018, *p* = 0.037). HPV status was not independently related to OS or PFS, a finding that was unchanged even when p16 was removed from the Cox analysis (*p* > 0.05; data not shown).

Data are summarized in Tables 2 and 3.

**Table 2:** Survival analysis – OS (126 patients)

Parameter	Distribution	Univariate	Multivariate
Age		*p* = 0.234	NE
≤ 60	82		
> 60	44		
Tumor diameter		***p* = 0.013**	p = 0.545
≤ 4 cm	102		
> 4 cm	24		
Lymphovascular invasion^a^		***p* < 0.001**	***p* = 0.001**
Yes	17		
No	108		
Lichen sclerosus ^b^		***p* = 0.019**	*p* = 0.286
Yes	37		
No	87		
Pathological tumor-free margin ^c^		*p* = 0.534	NE
Yes	108		
No	9		
Lymph node metastasis (yes vs. no)^d^		***p* < 0.001**	*p* = 0.469
Yes	30		
No	84		
Lymph node metastasis (number) ^d^		***p* < 0.001**	*p* = 0.166
> 1	10		
1	20		
0	84		
p16 ^e^		***p* = 0.007**	***p* = 0.02**
Positive	49		
Patchy/negative	63		
p53 ^f^		***p* = 0.012**	*p* = 0.987
Aberrant	61		
Wild-type	55		
HPV status ^g^		***p* = 0.021**	*p* = 0.197
Positive	66		
Negative	49		
Post-operative radiation		***p* < 0.001**	*p* = 0.985
Yes	29		
No	97		

Values in bold are statistically significant (*p*<0.05)

*Abbreviations*: *NE* not entered

^a^ Available for 125 patients

^b^ Available for 124 patients

^c^ By carcinoma; Available for 117 patients; remaining cases operated at other hospitals and specimens were inconclusive with respect to margin

^d^ Available for 114 patients

^e^ Data for 112 patients; remaining cases operated at other hospitals with block unavailable (*n* = 10) or stained with inconclusive result (*n* = 4)

^f^ Data for 116 patients; remaining cases operated at other hospitals with block unavailable (*n* = 10)

^g^ Data for 115 patients; remaining cases operated at other hospitals with block unavailable (*n* = 10) and 1 failed test

**Table 3:** Survival analysis – PFS (126 patients)

Parameter	Distribution	Univariate	Multivariate
Age		*p* = 0.859	NE
≤ 60	82		
> 60	44		
Tumor diameter		***p* = 0.038**	*p* = 0.982
≤ 4 cm	102		
> 4 cm	24		
Lymphovascular invasion^a^		***p* = 0.003**	***p* = 0.018**
Yes	17		
No	108		
Lichen sclerosus ^b^		***p* = 0.004**	*p* = 0.14
Yes	37		
No	87		
Pathological tumor-free margin ^c^		*p* = 0.295	NE
Yes	108		
No	9		
Lymph node metastasis (yes vs. no)^d^		***p* < 0.001**	*p* = 0.43
Yes	30		
No	84		
Lymph node metastasis (number) ^d^		***p* < 0.001**	*p* = 0.273
> 1	10		
1	20		
0	84		
p16 ^e^		***p* = 0.004**	***p* = 0.037**
Positive	49		
Patchy/negative	63		
p53 ^f^		*p* = 0.058	*p* = 0.464
Aberrant	61		
Wild-type	55		
HPV status ^g^		***p* = 0.039**	*p* = 0.325
Positive	66		
Negative	49		
Post-operative radiation		***p* < 0.001**	*p* = 0.674
Yes	29		
No	97		

Values in bold are statistically significant (*p*<0.05)

*Abbreviations*: *NE* not entered

^a^ Available for 125 patients

^b^ Available for 124 patients

^c^ By carcinoma; Available for 117 patients; remaining cases operated at other hospitals and specimens were inconclusive with respect to margin

^d^ Available for 114 patients

^e^ Data for 112 patients; remaining cases operated at other hospitals with block unavailable (*n* = 10) or stained with inconclusive result (*n* = 4)

^f^ Data for 116 patients; remaining cases operated at other hospitals with block unavailable (*n* = 10)

^g^ Data for 115 patients; remaining cases operated at other hospitals with block unavailable (*n* = 10) and 1 failed test

Univariate survival analysis was additionally performed for patients with HPV-negative and HPV-positive tumors, in the aim of comparing the prognostic role of LVSI in each group. In this analysis, as in analysis of the entire cohort, LVSI was associated with poor OS and PFS, with stronger association for patients with HPV-negative tumors (*p* = 0.003 and *p* = 0.007 for OS and PFS, respectively; Supplementary Figs. 1-A, 1-B) compared to those with HPV-positive tumors (*p* = 0.023 and *p* = 0.02 for OS and PFS, respectively; Supplementary Figs. 1-C, 1-D).

No significant differences were observed in comparative analysis of patients with limited LVSI (< 4 vessels) compared to those with extensive LVSI (≥ 4 vessels) in analysis of the entire cohort, with survival being in fact shorter for patients with fewer involved vessels (*p* = 0.278 and *p* = 0.259 for OS and PFS, respectively; Supplementary Figs. 1-E, 1-F).

## Discussion

The present study analyzed the presence and prognostic role of clinicopathologic parameters and biomarkers in a cohort of patients diagnosed with vSqCC and treated at a tertiary cancer center.

Numerous studies have assessed the prognostic role of clinicopathologic parameters in vulvar cancer [reviewed in 3], and the present discussion focuses on those that have additionally analyzed p16, p53 and/or HPV status. The latter group has reported widely discrepant results, reflecting differences in cohorts owing to epidemiology, patient age and case selection [9–19]. The parameters analyzed also differ across studies, as does the cut-off for some parameters (e.g., tumor diameter cut-off at 2 vs. 4 cm). Finally, definition of p16 expression as surrogate for HPV infection vs. molecular analysis of HPV status is another feature rendering comparisons difficult.

Among clinical parameters, lymph node metastasis and post-operative radiotherapy were strong prognosticators in univariate analysis of both OS and PFS, with less robust, though still significant association observed for tumor diameter, and no such role for age or BMI. With the exception of age, the present data are in agreement with previous reports [9, 11, 14, 16, 17, 19]. Older age has been reported to be associated with poor outcome in the majority [11–14, 17], though not all [16], studies. Of note, the cut-off applied for grouping patients in this category differs across study, being 78 years in the Alonso series [11], 70 years in the Kortekass series [17] and 65 years in the Barlow series [16], compared to 60 years in the present study, a fact that may explain the discrepancy.

Among morphological parameters, the presence of LVSI and LS, but not surgical resection involvement by VIN or carcinoma, were related to OS and PFS. The finding regarding LVSI is in agreement with several previous reports [14, 17, 19], though it was not observed in the Barlow series [16]. The prognostic role of LS has not been assessed in the studies discussed here, but LS has been reported to be associated with shorter PFS in several studies [reviewed in 3. Resection margin involvement by VIN and/or carcinoma has been reported to be associated with survival in some [14, 17], but not all [11] studies.

HPV detection rates in vSqCC have been reported to be as low as 19.4% [11] or 23% [20], and as high as 68.8% [21]. The data in the present study, in which 57% of tumors harbored HPV DNA, are within this range, but closer to the values in the latter series. As in other series [9–11, 13], HPV16 was the most frequently detected type, followed by HPV33 and HPV18. Our data are well in agreement with a recent large Danish study, in which HPV was detected in 52% of 1,308 vSqCC, of which the majority harbored high-risk HPV types, predominantly, in that order, types 16, 33 and 18 [22].

Good, but not full, agreement was observed between HPV status and p16 expression, supporting previous observations that while p16 may inform on HPV infection in the majority of cases [13], it cannot fully replace HPV typing and may best fit resource-limited conditions.

In the present study, p16 and p53 protein expression and HPV status were all significantly related to OS, whereas only p16 and HPV status were associated with PFS in univariate analysis. Multivariate analysis identified p16 expression and LVSI as independent prognosticators of both OS and PFS.

Few studies have assessed the relative prognostic value of p16, p53 and HPV in vSqCC. Our data are fully discordant with those of Alonso et al. [11]. In the latter study, p16, p53 and HPV were unrelated to OS or DFS, and comparable survival was observed for patients with HPV-associated and HPV-independent carcinomas. However, with only 19.4% of 98 tumors being HPV-positive, this study may have been underpowered to investigate this question. Data documenting better survival for patients with HPV-associated carcinomas have since been published by other groups [10, 18]. Several papers have reported significant association between p16 expression and DFS/PFS, OS or both, though HPV analysis has not been performed in these studies [13–16, 19]. A prognostic role for p16 and p53 separately [12] or for the combination p16/p53 [17] was reported in other studies.

An independent prognostic role for p16 and LVSI was identified in this study in analysis of both PFS and OS. p16 was an independent prognostic marker of OS in the Dong series [12], and the combination p16/p53 was independently related to RFS in the Kortekaas series [17]. However, our data are in best agreement with the results of Gadducci et al. [19], who recently reported on an association between p16 expression and LVSI in multivariate analysis, in a series of 78 vSqCC.

The lack of independent prognostic role for parameters that performed strongly in univariate analysis, such as lymph node metastasis and administration of radiotherapy, may be related to a relatively small number of events, particularly since cases included in multivariate analysis are only those who have all values entered (*n* = 105 in the present series).

Of note, LVSI was associated with poor OS and PFS also in separate analysis of patients with HPV-negative and HPV-positive tumors, despite the small number of events in each group (*n* = 8), with stronger association in the HPV-negative group. Conversely, the number of vessels involved does not appear to be informative of outcome in this cohort, based on analysis of the entire group.

In conclusion, analysis of 126 vSqCC highlighted the role of p16 IHC and LVSI in predicting outcome in this disease. Whether the role of p16 as an independent prognosticator is reproducible in series which have different proportion of HPV-negative and -positive carcinomas remains to be established in other cohorts. However, despite the fact that p16 staining is not informative of all HPV-positive carcinomas, inclusion of these 2 tests for prognostic purposes in everyday practice appears mandated based on our series.

### Supplementary Information

Below is the link to the electronic supplementary material.

**Supplementary Fig. 1. Additional analyses of LVSI**.** A:** Kaplan-Meier survival curve showing the association between the presence of LVSI and OS for 48 vulvar SqCC patients with HPV-negative tumors (1 patient with no LVSI data). Patients with LVSI (*n *= 8; red line) had mean OS of 44 months compared to 115 months for patients whose tumors did not have LVSI (*n *= 40, blue line; *p *= 0.003).** B:** Kaplan-Meier survival curve showing the association between the presence of LVSI and PFS for 48 vulvar SqCC patients with HPV-negative tumors (1 patient with no LVSI data). Patients with LVSI (*n *= 8; red line) had mean PFS of 41 months compared to 114 months for patients whose tumors did not have LVSI (*n *= 40, blue line; *p *= 0.007).** C:** Kaplan-Meier survival curve showing the association between the presence of LVSI and OS for 66 vulvar SqCC patients with HPV-positive tumors. Patients with LVSI (*n *= 8; red line) had mean OS of 107 months compared to 151 months for patients whose tumors did not have LVSI (*n *= 58, blue line; *p *= 0.023).** D:** Kaplan-Meier survival curve showing the association between the presence of LVSI and PFS for 66 vulvar SqCC patients with HPV-positive tumors. Patients with LVSI (*n *= 8; red line) had mean PFS of 102 months compared to 141 months for patients whose tumors did not have LVSI (*n *= 58, blue line; *p *= 0.02).** E:** Kaplan-Meier survival curve showing the association between the number of vessels involved by SqCC and OS for 17 patients with LVSI (8 with HPV-negative tumor, 8 with HPV-positive tumor, 1 patient with inconclusive HPV status). Patients with tumors that had involvement of ≥4 vessels (*n *= 8; red line) had mean OS of 99 months compared to 54 months for patients whose tumors had involvement of <4 vessels (*n *= 9, blue line; *p *= 0.278).** F:** Kaplan-Meier survival curve showing the association between the number of vessels involved by SqCC and PFS for 17 patients with LVSI (8 with HPV-negative tumor, 8 with HPV-positive tumor, 1 patient with inconclusive HPV status). Patients with tumors that had involvement of ≥4 vessels (*n *= 8; red line) had mean PFS of 95 months compared to 50 months for patients whose tumors had involvement of <4 vessels (*n *= 9, blue line; *p *= 0.259). (PPTX 7536 KB)

## Data Availability

Raw data may be obtained from the corresponding author upon reasonable request.
