# Supplementary material for: Lymphovascular invasion and p16 expression are independent prognostic factors in stage I vulvar squamous cell carcinoma
Source: Virchows Arch. 2023 Oct 16;484(6):951–63. doi: 10.1007/s00428-023-03670-y (PMC11186959; doi:10.1007/s00428-023-03670-y)
Supplement: Supplementary file 1 — Supplementary Fig. 1. Additional analyses of LVSI. A: Kaplan-Meier survival curve showing the association between the presence of LVSI and OS for 48 vulvar SqCC patients with HPV-negative tumors (1 patient with no LVSI data). Patients with LVSI (n = 8; red line) had mean OS of 44 months compared to 115 months for patients whose tumors did not have LVSI (n = 40, blue line; p = 0.003). B: Kaplan-Meier survival curve showing the association between the presence of LVSI and PFS for 48 vulvar SqCC patients with HPV-negative tumors (1 patient with no LVSI data). Patients with LVSI (n = 8; red line) had mean PFS of 41 months compared to 114 months for patients whose tumors did not have LVSI (n = 40, blue line; p = 0.007). C: Kaplan-Meier survival curve showing the association between the presence of LVSI and OS for 66 vulvar SqCC patients with HPV-positive tumors. Patients with LVSI (n = 8; red line) had mean OS of 107 months compared to 151 months for patients whose tumors did not have LVSI (n = 58, blue line; p = 0.023). D: Kaplan-Meier survival curve showing the association between the presence of LVSI and PFS for 66 vulvar SqCC patients with HPV-positive tumors. Patients with LVSI (n = 8; red line) had mean PFS of 102 months compared to 141 months for patients whose tumors did not have LVSI (n = 58, blue line; p = 0.02). E: Kaplan-Meier survival curve showing the association between the number of vessels involved by SqCC and OS for 17 patients with LVSI (8 with HPV-negative tumor, 8 with HPV-positive tumor, 1 patient with inconclusive HPV status). Patients with tumors that had involvement of ≥4 vessels (n = 8; red line) had mean OS of 99 months compared to 54 months for patients whose tumors had involvement of <4 vessels (n = 9, blue line; p = 0.278). F: Kaplan-Meier survival curve showing the association between the number of vessels involved by SqCC and PFS for 17 patients with LVSI (8 with HPV-negative tumor, 8 with HPV-positive tumor, 1 patient with inconclusive HP [file 428_2023_3670_MOESM1_ESM.pptx]

## Slide 1
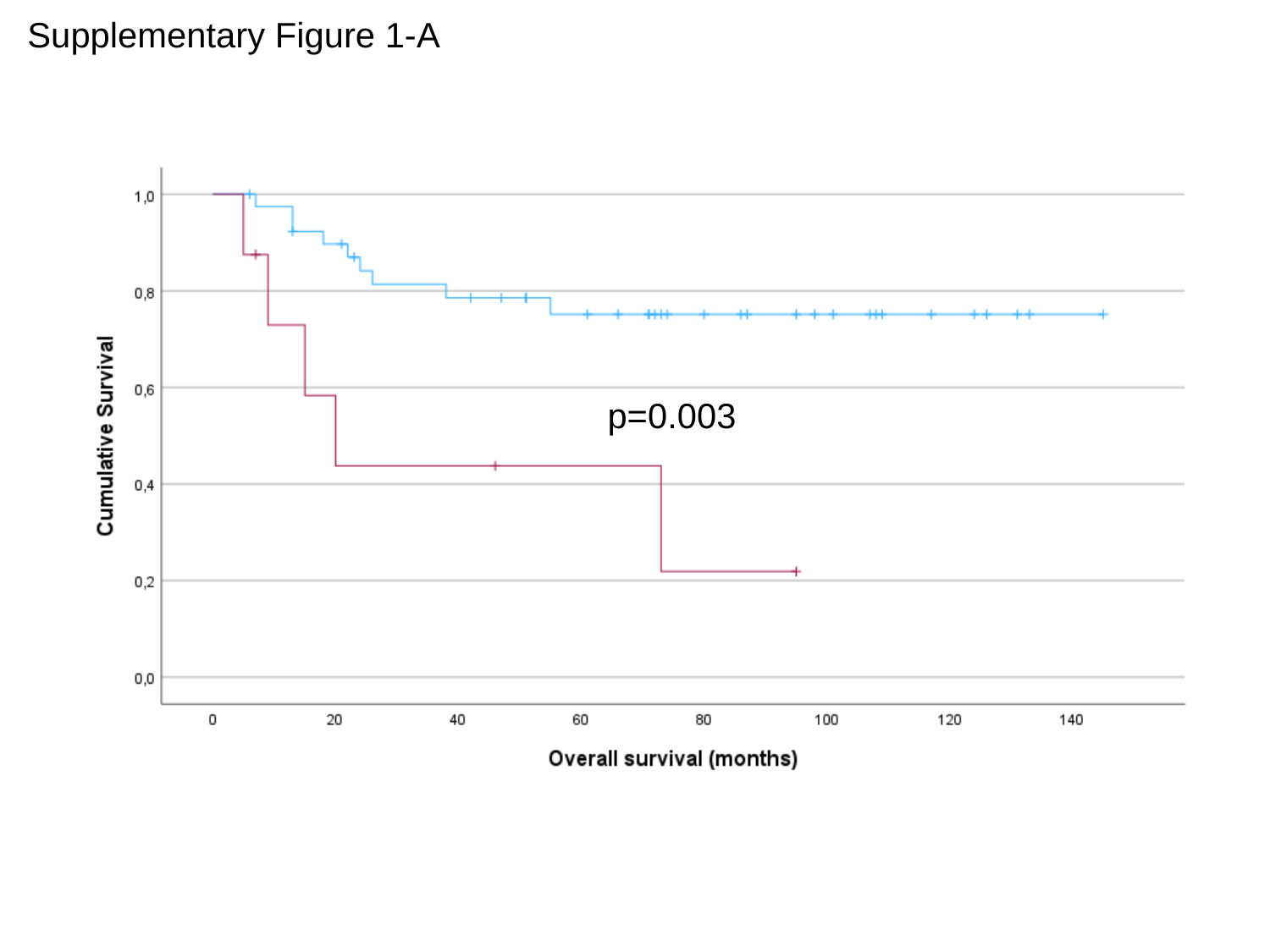

Supplementary Figure 1-A
p=0.003

## Slide 2
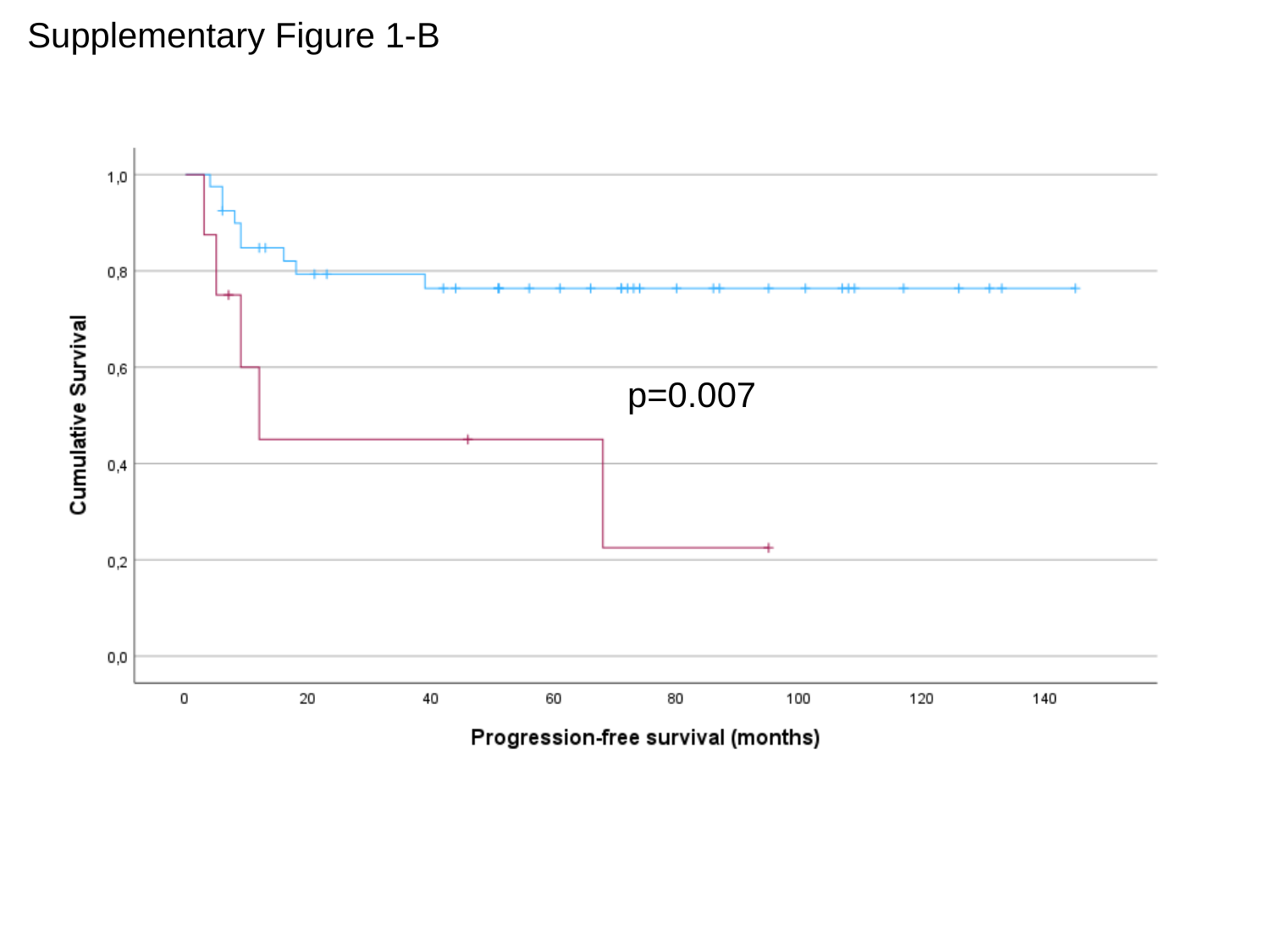

Supplementary Figure 1-B
p=0.007

## Slide 3
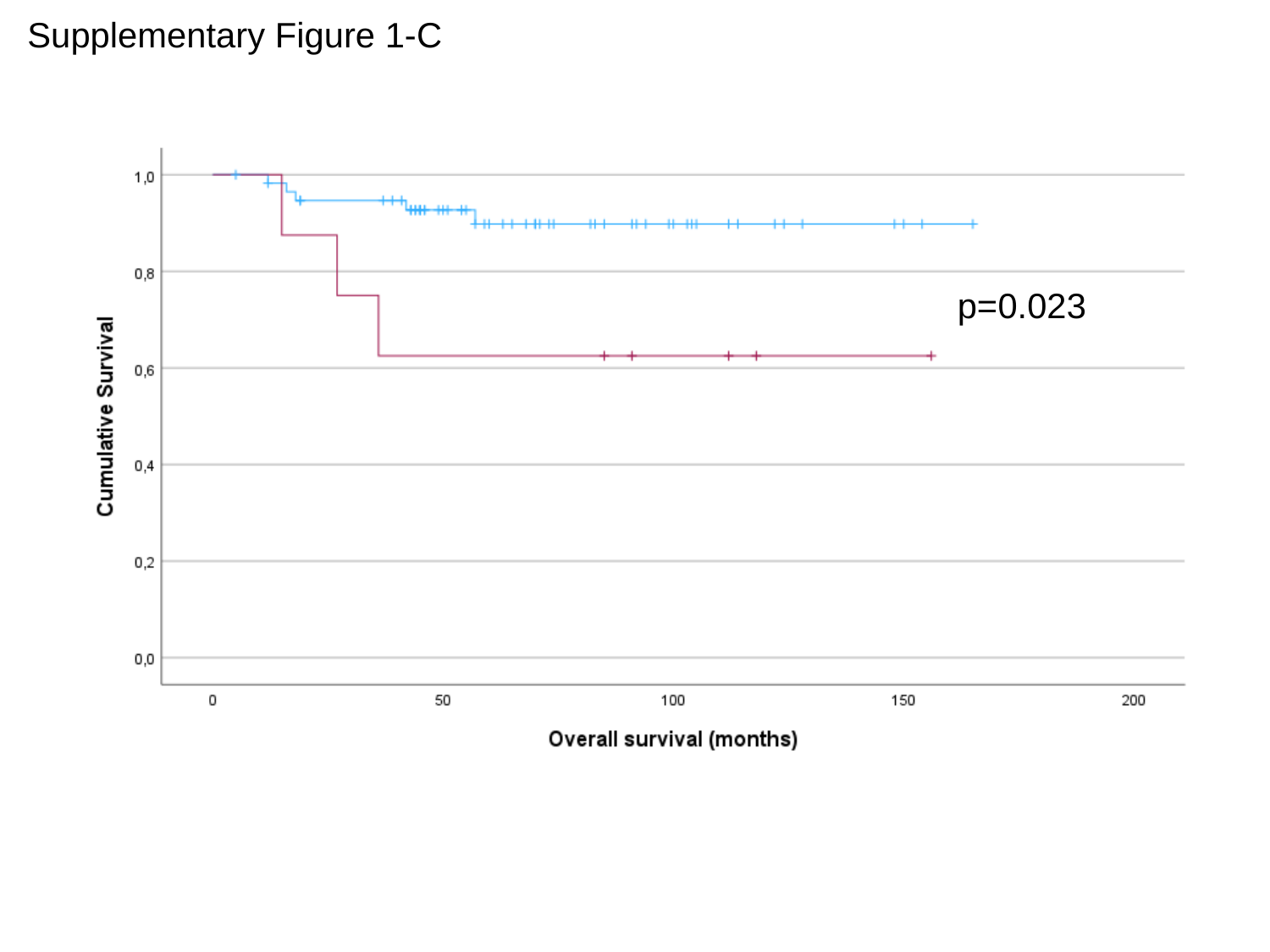

Supplementary Figure 1-C
p=0.023

## Slide 4
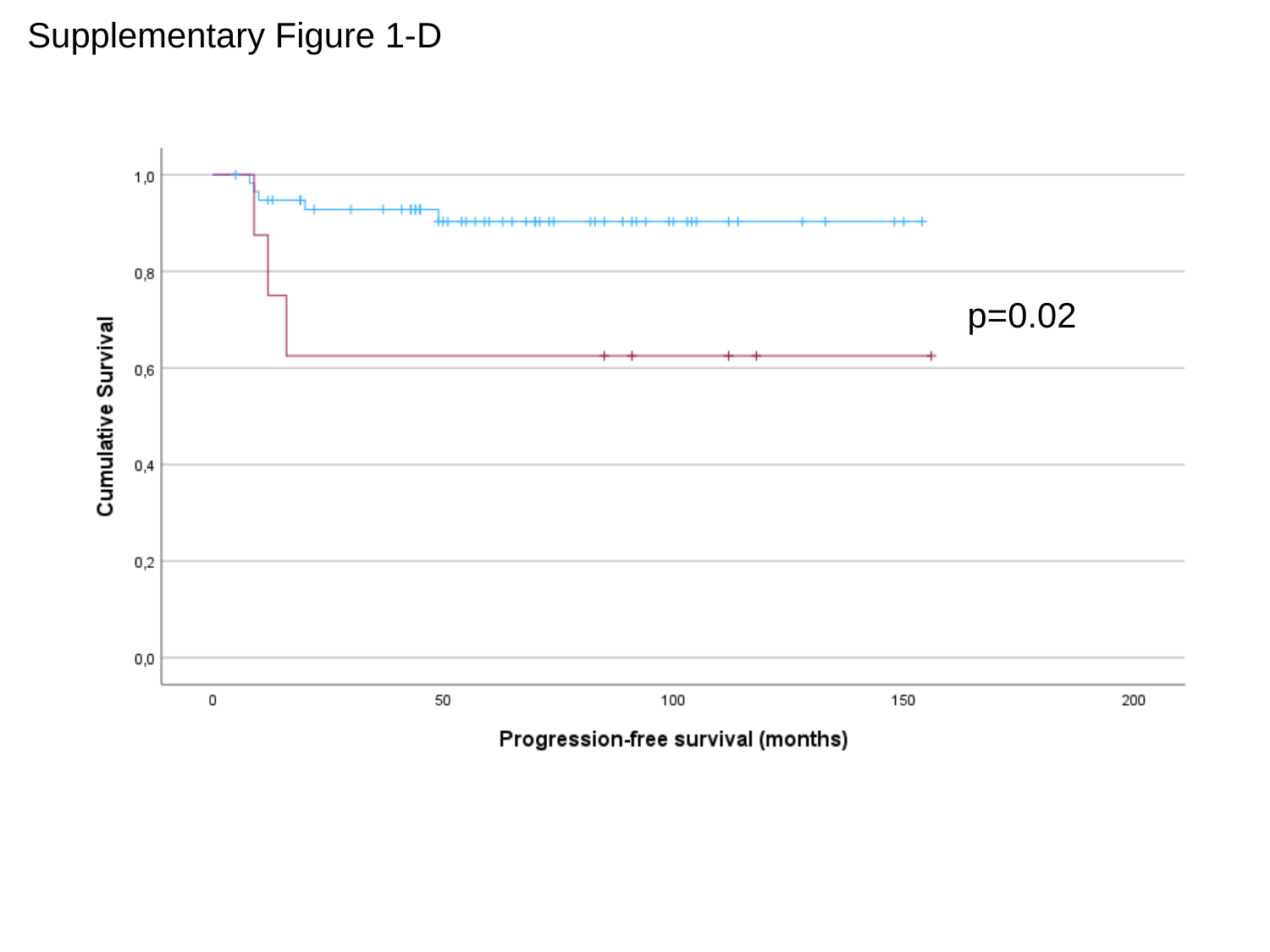

Supplementary Figure 1-D
p=0.02

## Slide 5
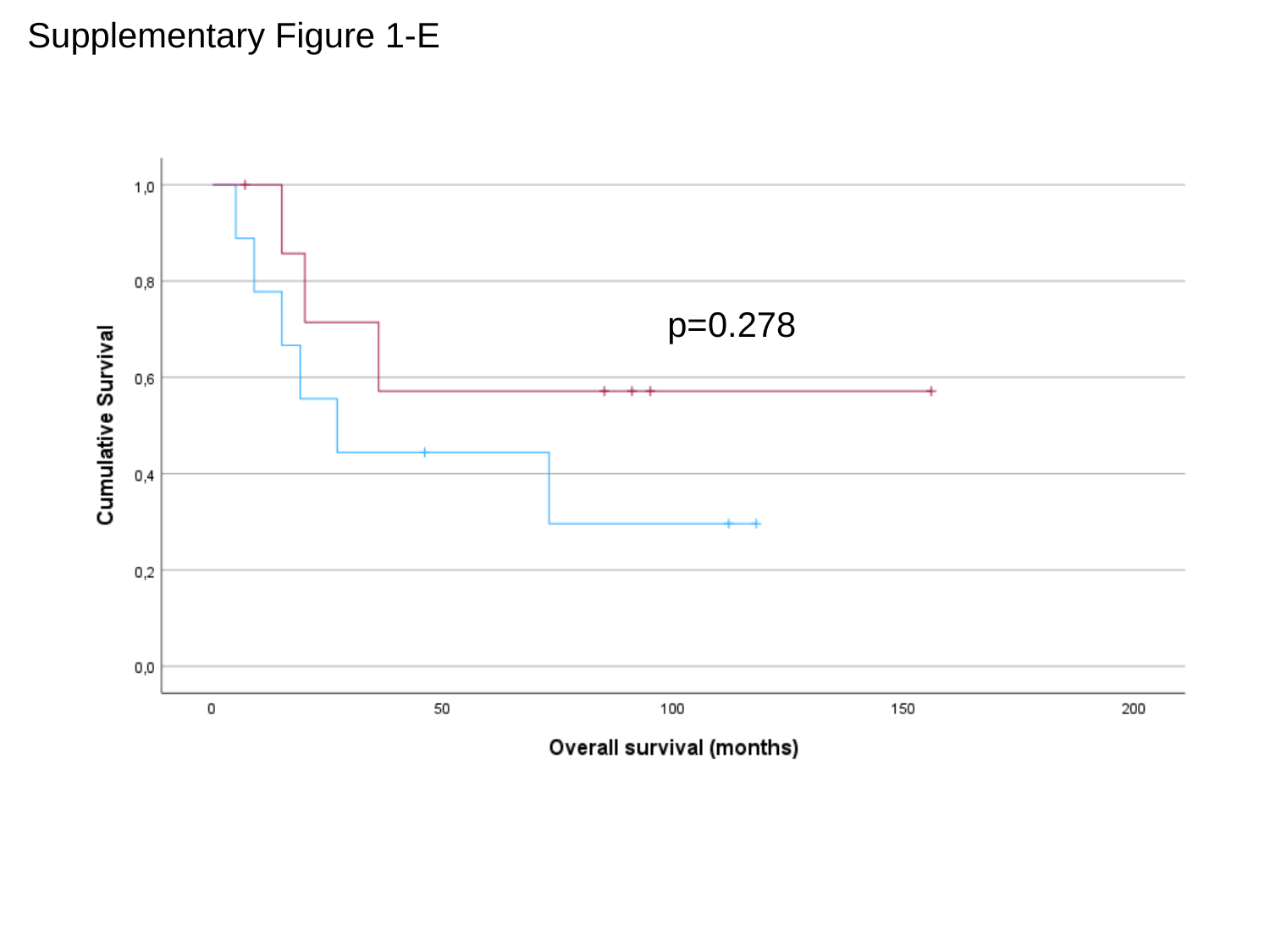

Supplementary Figure 1-E
p=0.278

## Slide 6
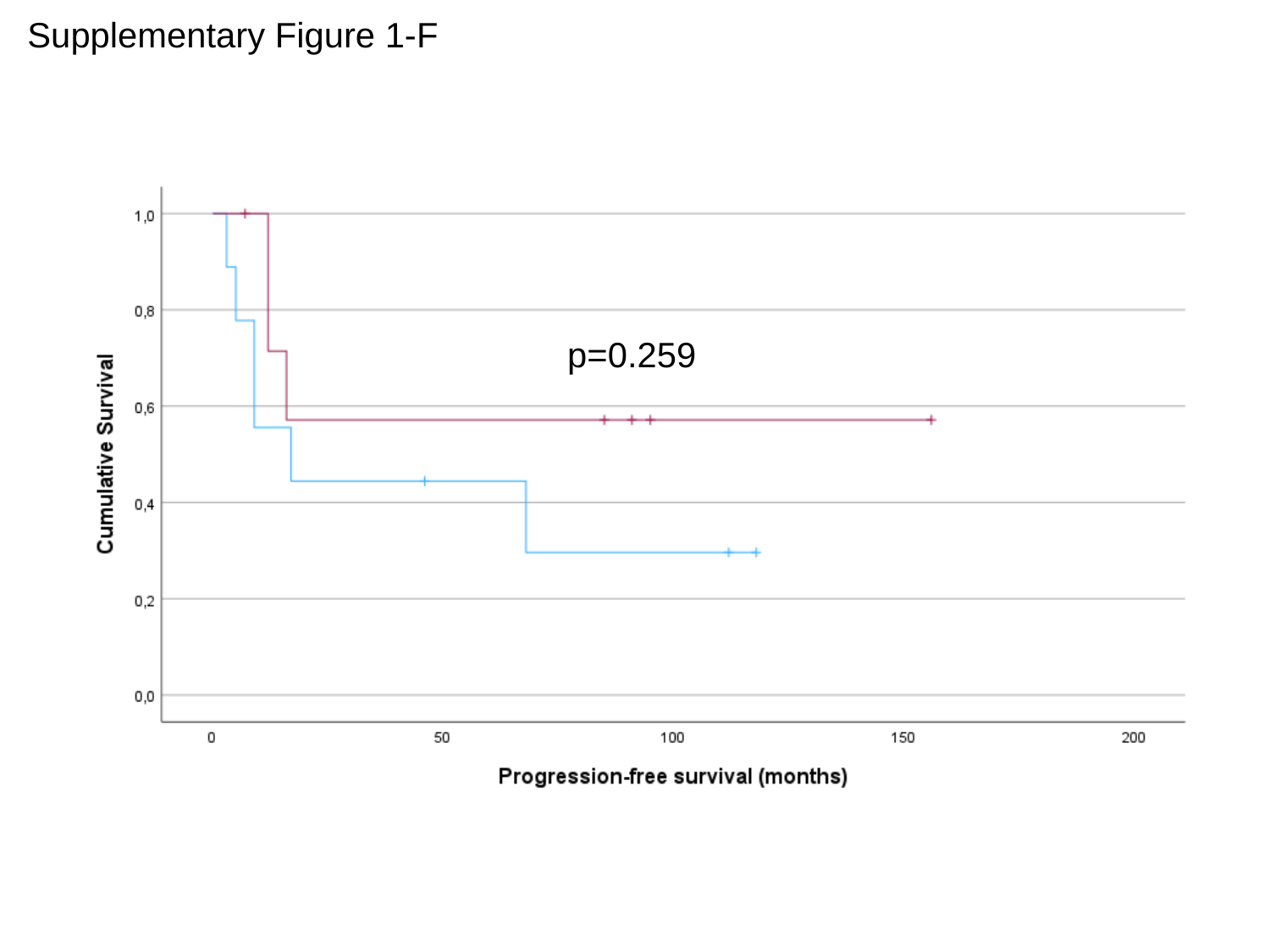

Supplementary Figure 1-F
p=0.259
